# Changes in vegetation structure and composition of a lowland mire over a sixty‐five‐year interval

**DOI:** 10.1002/ece3.6984

**Published:** 2020-11-12

**Authors:** Alexander T. Lovegrove, Adrian C. Newton, Paul M. Evans, Anita Diaz, Arthur C. Newton, Lynn Davy, Palmer J. Newbould

**Affiliations:** ^1^ Centre for Ecology, Environment and Sustainability Faculty of Science and Engineering Bournemouth University Poole UK; ^2^ College of Life and Environmental Sciences University of Exeter Penryn UK; ^3^ Wimborne UK; ^4^ Cirencester UK

**Keywords:** biodiversity, bog, conservation, determinants of plant community diversity and structure, environmental change, peatland, plant distribution

## Abstract

1. Mires are characterized by plant communities of high conservation and societal value, which have experienced a major decline in area in many parts of the world, particularly Europe. Evidence suggests that they may be particularly vulnerable to changes in climate and nutrient addition. Although they have been the focus of extensive paleoecological research, few attempts have been made to examine the dynamics of mire vegetation during the current era of anthropogenic environmental change.
2. To assess long‐term change in the spatial structure and composition of a lowland mire community, in 2016 we resurveyed plots first surveyed in 1951. Measures of species richness and composition were compared between the two surveys, and changes in community composition were related to plant traits.
3. Overall, mean species richness declined by 26%. The area of occupancy declined in 37% of species, which were primarily oligotrophic species typical of nutrient‐poor bog communities. Conversely, occupancy increased in 21% of species, especially those that were more tolerant of higher nutrient availability. These changes were associated with variation in plant functional traits, as indicated by an increase mean Ellenberg trait values for nitrogen and mean temperature, and a decline in values for precipitation. These results suggest that eutrophication and climate change have been key drivers of floristic change on this site.
4. *Synthesis*. This investigation provides a rare assessment of the dynamics of a mire community over a multi‐decadal interval. Results indicate that substantial change has occurred in the composition of the community, and the distribution of species within it. The investigation provides evidence of the impact of environmental change on the composition and structure of a lowland mire community, and highlights challenges for its future conservation.

## Graphical abstract

We assessed the long‐term change in the spatial structure and composition of a lowland mire community, by resurveying plots originally created in 1951. Results indicate that substantial change has occurred in the composition of the community, and the distribution of species within it. The investigation provides evidence of the impact of environmental change on the composition and structure of a lowland mire community, and highlights challenges for its future conservation.

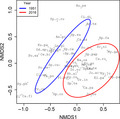

## 1 INTRODUCTION

Mires are wetland ecosystems characterized by the active accumulation of organic matter (peat) and are globally recognized as being important for biodiversity conservation, climate regulation, and human welfare (Parish et al., 2008). Mires extend over some 4 million km^2^ worldwide, occurring in more than 180 countries and accounting for more than a third of the world's wetland area, while being especially abundant in boreal and subarctic regions (Joosten & Clarke, 2002; Parish et al., 2008). Human activities including peat cutting, drainage, and conversion to agriculture and forestry have led to major losses of mire ecosystems in many parts of the world. The largest declines in area have been recorded in Europe, where less than 50% of the original mire extent now remains (Joosten & Clarke, 2002). Mire ecosystems are also vulnerable to environmental pressures such as climate change and eutrophication. For example, in northern Europe deposition of atmospheric nitrogen can lead to a decline in characteristic *Sphagnum* species and their replacement by more nitrophilous mosses, nitrogen‐dependent grass species such as *Molinia caerulea* and *Deschampsia flexuosa*, and tree species such as *Betula pubescens* (Bobbink et al., 2008). Climate change can result in changes to the water balance of peatlands, which can increase desiccation and the decomposition rate of accumulated organic matter, leading to an increased frequency of fires (Dieleman et al., 2016; Turetsky et al., 2015).

Vegetation changes occurring in mire ecosystems could potentially have significant impacts on human society, by influencing the provision of ecosystem services to people. Most significant among these services is the important role that mires play in regulating the climate at both local and global scales, by acting as a major sink of atmospheric carbon and a source of greenhouse gases including carbon dioxide, methane, and nitrous oxide (Bonn et al., 2014; Parish et al., 2008). Northern peatlands are considered to be the most efficient terrestrial ecosystem for storing carbon when assessed per unit area, and currently account for around 30% of the Earth's terrestrial carbon store (Dieleman et al., 2016). Mires also have a significant influence on the hydrology of catchments, by affecting water storage, water quality, and flood mitigation (Joosten & Clarke, 2002; Parish et al., 2008). The provision of many of these services is dependent on the process of peat formation, which occurs following the submergence of organic matter. Mires are typically characterized by a complex vertical structure featuring a variety of different microforms, ranging from wet pools or hollows to drier mounds or hummocks (Belyea & Clymo, 2001). These different structural features respond dynamically to changing environmental conditions, such as the position of the water table. For example, hollows expand laterally during wet periods, whereas hummocks increase during drier periods (Belyea & Clymo, 2001). The long‐term rate of peat formation can therefore be explained by feedbacks between the biological and hydrological processes occurring near the surface of the mire (Belyea & Clymo, 2001; van Breemen, 1995). Examples of such feedbacks include (Limpens et al., 2008): (a) trade‐offs between hummock‐ and hollow‐inhabiting *Sphagnum* species, with slower decomposition by the former and higher productivity among the latter; (b) lower decomposition rates of *Sphagnum* species compared with most coinhabiting vascular plant species; (c) lower litter C:N ratio increasing the decomposition rate following nitrogen deposition; and (d) species composition influencing the structure and hydraulic conductivity of peat, which may then influence the growth and survival of plant species.

Understanding the extent to which these processes are occurring in mire ecosystems depends on analyzing how the composition and structure of mire vegetation responds to environmental changes over the long term. The primary evidence for such long‐term dynamics is provided by the numerous paleoecological studies that have been conducted in peatlands (Payne et al., 2016). Recent research has tended to focus on assessing peatland development and the impacts of climate change during the late Holocene, employing techniques including palynology, macrofossil analysis, testate amoebae, isotopes, and biomarkers (Blundell & Holden, 2015). Such research has made important contributions to understanding the response of mire communities to changing climate, as well as the role of successional processes and species interactions in long‐term community dynamics (Heijmans et al., 2008; Mauquoy, et al., 2002; Mauquoy et al., 2002). Results have also indicated that mire communities can demonstrate pronounced stability; for example, through analysis of peat stratigraphy, Rydin and Barber (2001) demonstrated that local coexistence of *Sphagnum* species can persist over timescales of several centuries. However, the value of paleoecological approaches for understanding the processes influencing vegetation dynamics is limited by the lack of spatial, temporal, and taxonomic precision (Jackson & Blois, 2015). Paleoecological evidence has been complemented by a number of experimental studies designed to examine the response of mire communities to different types of environmental change, including temperature, atmospheric CO_2_ concentration, water level, and nitrogen deposition, which have been shown to influence the relative abundance of species and the functioning of mire ecosystems (Heijmans et al., 2008). Although experimental investigations can provide valuable insights into the mechanisms underpinning vegetation dynamics, such studies are necessarily short‐term, typically being undertaken over three or four growing seasons.

Very few studies of mire vegetation have been undertaken over multi‐decadal timescales during the current era of anthropogenic change. It is therefore unclear whether the changes in vegetation predicted by short‐term experiments actually occur in field situations over longer time periods (Hájková et al., 2011). Here, we examine changes in the plant communities associated with a mire located in southern England, assessed over an interval of 65 years. Cranes Moor is a valley mire site located in the New Forest National Park that was originally surveyed by Palmer Newbould in 1951 as part of his PhD research (Newbould, 1953, 1960). As part of this research, a group of species was systematically mapped across the entire site, providing a unique opportunity to assess distributional changes of these species over an interval of 65 years. These observations were supported by complete inventories of species present in survey plots established in each of the vegetation types present (Newbould, 1953, 1960), enabling changes in species composition also to be assessed. Here, we present the results of a resurvey of the vegetation of Cranes Moor, using the same design and approaches adopted in the 1951 survey. The specific objectives were to test whether any changes had occurred in the abundance and distribution of the species that were surveyed across the site, and also to examine which factors were responsible for any differences observed. Previous research into the long‐term changes in plant communities in the neighboring county of Dorset has highlighted eutrophication and climate change as key drivers of vegetation change during the past 70 years (Diaz et al., 2013; Keith et al., 2009, 2011; Newton et al., 2012). On this basis, we hypothesized that the composition of plant communities in Cranes Moor would have similarly been influenced by these factors, leading to a decline in species associated with low nutrient status, and those susceptible to summer drought.

## 2 MATERIALS AND METHODS

### 2.1 Study area

The site is described by Newbould (1960) and by Grant et al. (2014), from which the following account is derived. Cranes Moor is located on the western edge of the New Forest National Park and is part of one of the largest mire systems in southern England (50.824°N, 1.726°W; OS SU194028) (Figure S1). The mire lies in a basin, the surface geology of which is largely composed of Palaeogene Chama and Becton Sand Formations, which are characterized by podsol development under surrounding areas of heathland. The heathland is dominated by *Calluna vulgaris,* which occurs with *Pteridium aquilinum*, *Ulex europaeus*, *Ulex minor,* and *Erica cinerea* in areas of dry heath, and abundant *Erica tetralix* in wetter areas. There are some areas of closed‐canopy pinewood (*Pinus sylvestris*) near to the mire, which originate from 19th‐century plantations and are periodically cleared from the heathland and mire sites by the Forestry Commission. *Betula pendula* is also locally present around the fringes of the mire. In addition to dry and wet heath communities, Newbould (1960) describes an extensive area of bog over which *Schoenus nigricans* is abundant, areas where *Myrica gale* and *M. caerulea* are dominant, and other areas where *Rhynchospora alba* and *Sphagnum papillosum* are abundant (Figure S2). Cranes Moor has been the focus of significant paleoecological research interest (Grant et al., 2009, 2014; Seagrief, 1960); it is unusual among lowland mires in providing a record of *Sphagnum* peat deposits from the early Holocene (c. 10,500 BP) (Grant et al., 2014). Evidence from peat stratigraphy suggests that the surface vegetation is likely to have been largely or solely ombrogenous from the early Holocene onwards, although the sequence has been disturbed by recent peat cutting (Grant et al., 2014).

### 2.2 Data collection

#### 2.2.1 The “Newbould Survey”, 1951

In 1960, Palmer Newbould published an extensive study of the vegetation and abiotic characteristics of Cranes Moor, based on a survey that he undertook in 1951 (Newbould, 1953, 1960). This study involved mapping the distributions of 19 plant species across the entire surface of the mire, using a sampling grid of 1,611 cells each with dimensions of 15 × 15 m. The abundance of each species within each grid cell was subjectively recorded based on a scale of absent, occasional, frequent, or abundant. Additionally, 13 “vegetation plots” of 20 × 20 m were placed in selected areas of representative vegetation types, and within these, the presence of all vascular plant and bryophyte species was recorded. Records were made by surveying 50 randomly distributed 25 × 25 cm quadrats in each of these vegetation plots (Figures S1 and S2).

#### 2.2.2 Resurvey, 2016

We resurveyed Cranes Moor bog following the original methodology used by Newbould (1960). The survey area was located by using digital scans of maps from the original publication, which were subsequently georeferenced in ArcGIS 10.3 (ESRI Software). Landmark features, including road intersections, bridges, and railway tracks, were used where they could be clearly located on the original map and on a spatially referenced OS map of the New Forest. Vegetation types or features were not used for any part of this process. Once the outline of the survey area was referenced, a network of 15 × 15 m grid cells was overlaid onto the map using the fishnet function in ArcGIS to form the basis of a new survey.

The vegetation was surveyed during July–August 2016. Grid cells were located in the field using a GPS handheld device (GPS Map 64, Garmin) with the corners of each cell loaded as points. The abundance of the selected species was recorded subjectively using Newbould's scale of absent, occasional, frequent, or abundant, coded as 0–3. Additional species were also recorded compared to the original survey (Table S8). The 13 vegetation plots were also relocated using GPS, with the abundance of all vascular plant and bryophyte species recorded using the same methodology as Newbould (1960). Some taxonomic changes have occurred since the original study, so to avoid confusion the nomenclature of all taxa followed Stace (2010) and the original names in Newbould's survey have been updated (Table S8).

### 2.3 Data analysis

All analyses were conducted in R version 3.3.2 (R Core Team, 2016).

#### 2.3.1 Vegetation maps

Vegetation maps were produced in ArcGIS 10.3 (ESRI Software) by recording the abundances of species in each grid cell (hereafter referred to as “landscape survey”). This was undertaken using a scale where 0 = absent and 3 = abundant, which then was used to assess the distribution of each species. Newbould's original maps were digitized in ArcGIS with the grid cells overlaid, and enabling the abundance of each species in each grid cell to be recorded. This permitted assessment of the changes in species abundance and distribution between the two surveys.

#### 2.3.2 Changes in species distributions and composition

The area of occupancy (IUCN, 2001) was calculated for species recorded in both the 2016 landscape survey and Newbould's original study, by counting the number of grid cells within which each species was present. We tested the significance of changes in the area of occupancy for each species using McNemar's test (McNemar, 1947). All assumptions for this test were met. Data from the 13 vegetation plots were then compared to the dataset obtained by Newbould, to examine the difference in species composition. The significance of any change in species richness at the scale of individual plots (i.e., ± α‐diversity) between surveys was tested using a paired *t* test. Paired *t* tests or Wilcoxon rank‐sum tests were used to determine any significant decreases or increases in the abundance of individual species between 1960 and 2016. The choice of test depended on whether the data met the normality distribution assumption.

Community composition was further explored by subjecting the vegetation plot data to multivariate analysis using the vegan package in R, specifically using permutational multivariate analysis of variance (PERMANOVA) (Oksanen et al., 2016). This is a nonparametric multivariate statistical test that is used to compare groups of objects. This analysis was performed using the Bray–Curtis dissimilarity measure with 1,000 permutations, using the following formula after removing species that were present in fewer than three plots overall:

$${\mathrm{BC}}_{\mathrm{ij}}=1‐\frac{2C_{\mathrm{ij}}}{S_{i}+S_{j}}$$

where *S_i_* and *Sj* are the total number of specimens counted on plots *i* and *j,* respectively, and *C_ij_* is the sum of the lesser counts for only those species found in both plots.

The results were subsequently visualized in two‐dimensional space using nonmetric multidimensional scaling (nMDS; Everitt, 1978) employing the functions *metaMDS*, *ordiplot,* and *ordiellipse* in the vegan package of R, to enable visualization of changes in the sample plant community over time. Vegetation plot data were also analyzed using the MAVIS computer program (Smart et al., 2016), which provides a method for determining any change in National Vegetation Community (NVC) type (Rodwell, 1991) over time.

#### 2.3.3 Drivers of change

We selected different plant attributes to examine whether changes in species composition and abundance were associated with these traits. For this, we used PLANTATT and BRYOATT databases, which are collections of attributes on status, size, life history, geography, and habitats of British and Irish vascular plant species (Hill et al., 2004) and bryophytes (Hill et al., 2007), respectively. Attributes that were analyzed were the Ellenberg trait values for N (nitrogen), L (light), R (pH), and F (moisture), mean temperature in January (Tjan), mean temperature in July (Tjul), and mean annual precipitation amount (Prec). Mean Ellenberg trait values were calculated for each grid cell using the landscape survey data, and for each vegetation plot based on species composition. As the grid cell data were not normally distributed, Wilcoxon signed‐rank tests (with continuity correction) were used to determine the significance of a change in Ellenberg values over time. Vegetation plot data were normally distributed and were therefore analyzed using paired *t* tests with Welch's correction where variance was not homogeneous. Tests were also performed on the mean Ellenberg trait values of species that declined in abundance, from either the landscape survey or the vegetation plots, compared to those that did not decline over the survey interval.

## 3 RESULTS

### 3.1 Description of survey

For the landscape survey, ten grid cells could not be reached in 2016 as they were inaccessible, because of unstable substrate and deep water; a total of 1,601 were therefore surveyed. The missing data from the 10 grid cells were excluded from the analysis by removing data from both 1951 and 2016 (Figure S5). Nineteen species were mapped by Newbould in his original study, and these plus an additional 14 species were mapped in 2016 to examine distribution patterns (Table S8). One of the mapped species, *Hammarbya paludosa*, was not found in the 2016 survey. In the vegetation plots, Newbould recorded 48 plant and bryophyte species in 1951, compared to 50 species in the resurvey. In these plots, 11 plant species were recorded in 1951 but not in 2016, and 11 were recorded in 2016 but not in 1951 (Table S1).

### 3.2 Species distribution and abundance

Results of McNemar's test suggested that changes in the area of occupancy were large and statistically significant for 58% (11/19) of species, indicating shifts in spatial distribution (Table 1, Figures S3 and S4). Four species increased their area of occupancy since 1951, namely *P. aquilinum*, *Sphagnum cuspidatum*, *Sphagnum subnitens,* and *P. sylvestris* (sapling). Significant declines in the area of occupancy were observed in seven species, namely *Trichophorum germanicum*, *R. alba*, *Carex rostrata*, *Cirsium dissectum*, *Sphagnum compactum*, *Rhynchospora fusca,* and *Lycopodiella inundata*. Decline in occupancy of the latter two species was >90%. Patterns of changes in distribution differed markedly between species (Figure S5). *Pteridium aquilinum* largely spread from locations where it was present in 1951 into adjacent areas, particularly along the northern perimeter of the site. In contrast, *S. cuspidatum* and *P. sylvestris* colonized much of the central portion of the site, from which they were largely absent in the first survey. For *S. subnitens,* the converse was true; having originally been concentrated in central parts of the study area, it has become more widespread in peripheral areas over time.

**TABLE 1:** Change in the area of occupancy of species surveyed in 1951 and 2016

Species	Area occupied (ha)		Change in occupancy (ha)	Relative change (%)	*p*
	1951	2016			
*Pteridium aquilinum*	2.50	4.86	2.36	94.6	**<0.001**
*Sphagnum cuspidatum*	6.35	10.3	3.94	62.1	**<0.001**
*Sphagnum subnitens*	4.73	6.19	1.46	30.9	**0.001**
*Pinus sylvestris* sapling	10.4	11.9	1.53	14.7	**0.01**
*Pinus sylvestris* seedling	24.4	25.0	0.58	2.40	0.3
*Sphagnum magellanicum*	4.88	4.88	0	0	1
*Sphagnum papillosum*	22.7	22.6	−0.09	−0.40	0.88
*Schoenus nigricans*	9.81	9.70	−0.11	−1.15	0.81
*Myrica gale*	15.3	14.7	−0.68	−4.40	0.07
*Erica cinerea*	7.29	6.84	−0.45	−6.17	0.31
*Menyanthes trifoliata*	3.42	2.86	−0.56	−16.4	0.05
*Ulex europaeus*	2.86	2.27	−0.59	−20.5	0.06
*Trichophorum germanicum*	10.2	7.36	−2.88	−28.1	**<0.001**
*Rhynchospora alba*	26.2	18.8	−7.38	−28.2	**<0.001**
*Carex rostrata*	3.11	2.18	−0.92	−29.7	**<0.001**
*Cirsium dissectum*	4.97	2.95	−2.03	−40.7	**<0.001**
*Sphagnum compactum*	17.2	10.0	−7.25	−42.0	**<0.001**
*Rhynchospora fusca*	1.78	0.16	−1.62	−91.1	**<0.001**
*Lycopodiella inundata*	2.32	0.05	−2.27	−98.1	**<0.001**

Note

Results in bold indicate the difference was statistically significant at *p* < 0.05 (McNemar's test).

Similarly, contrasting changes in distribution patterns were observed in those species that declined over the survey interval. For example, the distribution of *T. germanicum* and *S. compactum* contracted in the peripheral parts of the site, whereas *R. alba*, *C. rostrata,* and *C. dissectum* declined more markedly within central areas (Figures S3–S5). Both *R. fusca* and *L. inundata* declined throughout their original ranges within the site, and both are now characterized by very restricted and patchy distributions. Some species displayed pronounced increases in local abundance, even though their overall distribution patterns remained largely unaltered; examples include *M. gale*, *Sphagnum magellanicum*, *S. papillosum,* and *S*. *nigricans* (Table 1 and Figure S5). This highlights the fact that distributional changes at the landscape scale were not necessarily linked to changing abundance at the local scale.

### 3.3 Species composition

Mean species richness within the 13 vegetation plots showed a significant decline from 1951 (mean ± *SE* values of 27.46 ± 1.15) to 2016 (20.38 ± 1.16, *p* = .001), indicating a change in community composition across the study area. Results of paired *t* tests and Wilcoxon tests suggested that changes in abundance were statistically significant for 29% (19/65) of species (Tables 2 and S6). Overall, 46 species stayed “stable” (i.e., did not decline or increase significantly). Significant declines in abundance were observed in 16 species (Table 2). The species that displayed the greatest reduction in abundance were *Kurzia pauciflora* and*Narthecium ossifragum*, which declined by 51% and 44%, respectively. The species that increased the most were *Sphagnum palustre*, *Hypnum jutlandicum,* and *Eleocharis palustris,* which were not recorded in 1951.

**TABLE 2:** Change in abundance of species surveyed in the vegetation plots in 1951 and 2016

Species	Mean 1951	*SE*	Mean 2016	*SE*	Mean Diff	Test statistic	*p*	+/−
						*t*‐value	*t* test	
*Calluna vulgaris*	50	8.27	24.31	3.63	−25.69	4.004	**0.002**	−
*Drosera rotundifolia*	50.15	6.40	44	7.75	−6.15	0.637	0.536	
*Eriophorum angustifolium*	88.46	4.18	64.92	4.9	−23.54	3.741	**0.003**	−
*Sphagnum denticulatum*	24.15	4.78	2.92	1.19	−21.23	4.355	**<0.001**	−
Species	**Mean 1951**	***SE***	**Mean 2016**	***SE***	**Mean Diff**	***W*‐value**	**Wilcox test**	
*Anagallis tenella*	3.08	2.33	0.92	0.62	−2.15	10	0.588	
*Aneura pinguis*	19.69	4.34	0.77	0.77	−18.92	66	**0.004**	−
*Aulacomnium palustre*	5.85	3.43	0	0	−5.85	10	0.1	
*Betula pendula*	0	0	0.15	0.15	0.15	0	1	
*Calypogeia azurea*	30.77	10.02	5.69	3.04	−25.08	66.5	**0.034**	−
*Campylopus flexuosus*	1.38	0.80	0	0	−1.38	10	0.098	
*Carex panicea*	2.92	1.39	10.31	5.46	7.38	12.5	0.26	
*Carex rostrata*	0.92	0.66	5.08	2.96	4.15	1.5	0.269	
*Carex viridula* subsp. *oedocarpa*	0	0	1.69	1.53	1.69	0	0.371	
*Cephalozia bicuspidata*	43.85	8.65	1.23	0.92	−42.61	91	**0.002**	−
*Cirsium dissectum*	8.31	5.39	0.77	0.53	−7.54	13.5	0.136	
*Cladonia crispata*	2.15	1.48	0	0	−2.15	3	0.371	
*Cladonia floerkeana*	7.08	4.88	0	0	−7.08	10	0.098	
*Cladonia portentosa*	28.92	8.35	2.46	0.91	−26.46	52.5	**0.012**	−
*Cladonia uncialis*	6.15	2.47	0.61	0.42	−5.54	34	**0.029**	−
*Dactylorhiza maculata*	0	0	0.31	0.31	0.31	0	1	
*Dicranum scoparium*	0	0	0.15	0.15	0.15	0	1	
*Diplophyllum albicans*	0.15	0.15	0	0	−0.15	1	1	
*Drosera intermedia*	4.46	3.06	5.38	2.89	0.92	14	0.339	
*Eleocharis palustris*	0	0	5.23	1.82	5.23	0	**0.022**	+
*Eleocharis quinqueflora*	0	0	0.31	0.21	0.31	0	0.346	
*Erica tetralix*	97.08	1.64	92	1.26	−5.08	68	**0.025**	−
*Gymnocolea inflata*	0.77	0.53	0.61	0.61	−0.15	3	1	
*Hypnum cupressiforme*	24.77	6.03	0.15	0.15	−24.61	78	**0.002**	−
*Hypnum jutlandicum*	0	0	5.23	1.80	5.23	0	**0.009**	+
*Hypogymnia physodes*	5.85	3.47	0	0	−5.85	21	**0.035**	−
*Juncus acutiflorus*	2.77	0.95	12.46	4.22	9.69	11	0.055	
*Juncus bufonius*	0	0	0.61	0.35	0.61	0	0.174	
*Juncus bulbosus*	0	0	0.31	0.31	0.31	0	1	
*Juncus compressus*	0	0	0.15	0.15	0.15	0	1	
*Kurzia pauciflora*	50.61	7.32	0	0	−50.61	91	**0.002**	−
*Leucobryum glaucum*	0.61	0.27	0.15	0.15	−0.46	6	0.149	
*Lycopodiella inundata*	0.15	0.15	0	0	−0.15	1	1	
*Menyanthes trifoliata*	0	0	0.46	0.46	0.46	0	1	
*Molinia caerulea*	97.54	1.65	95.08	1.25	−2.46	58	0.145	
*Myrica gale*	22.61	9.2	19.23	7.45	−3.38	15	0.402	
*Narthecium ossifragum*	85.69	7.45	41.85	5.07	−43.85	88	**0.003**	−
*Odontoschisma sphagni*	35.69	9.85	0.15	0.15	−35.54	66	**0.004**	−
*Pedicularis sylvatica*	0.61	0.35	1.38	0.83	0.77	7	0.524	
*Pinguicula lusitanica*	1.08	0.37	0.77	0.36	−0.31	21.5	0.66	
*Pinus sylvestris*	11.69	5.16	4.15	1.07	−7.54	35	0.474	
*Polygala serpyllifolia*	10.15	3.88	0.61	0.35	−9.54	36	**0.014**	−
*Potamogeton polygonifolius*	8.61	5.77	8.77	3.81	0.15	11.5	0.4	
*Potentilla erecta*	1.54	0.82	0	0	−1.54	10	0.098	
*Quercus robur*	0.15	0.15	0	0	−0.15	1	1	
*Rhynchospora alba*	47.08	10.96	38.46	9.19	−8.61	56.5	0.463	
*Schoenus nigricans*	15.85	9.77	14.31	6.69	−1.54	11	1	
*Sphagnum capillifolium*	6.92	2.27	2.61	1.46	−4.31	31	0.078	
*Sphagnum capillifolium* subsp. *rubellum*	0.77	0.48	0	0	−0.77	6	0.174	
*Sphagnum compactum*	25.85	11.09	6.61	4.67	−19.23	29	0.141	
*Sphagnum cuspidatum*	4	1.57	2	1.54	−2	26.5	0.261	
*Sphagnum fallax*	6.31	4.22	2.31	1.19	−4	10	0.59	
*Sphagnum inundatum*	11.38	4.21	0.77	0.77	−10.61	45	**0.009**	−
*Sphagnum magellanicum*	10.31	7.05	4	2.62	−6.31	11	1	
*Sphagnum palustre*	0	0	6	2.62	6	0	**0.022**	+
*Sphagnum papillosum*	52.46	11.55	29.85	8.77	−22.61	53.5	0.075	
*Sphagnum subnitens*	9.08	4.7	0.61	0.42	−8.46	24	0.106	
*Sphagnum tenellum*	26.77	10.86	14.77	4.3	−12	46	0.61	
*Trichophorum cespitosum*	2.31	1.22	0.61	0.61	−1.69	10	0.098	
*Ulex minor*	0.15	0.15	0	0	−0.15	1	1	
*Utricularia minor*	2	2	0	0	−2	1	1	

Note

Results in bold indicate a significant result. − indicates that the species declined; + indicates that the species increased in abundance.

**FIGURE 1:**
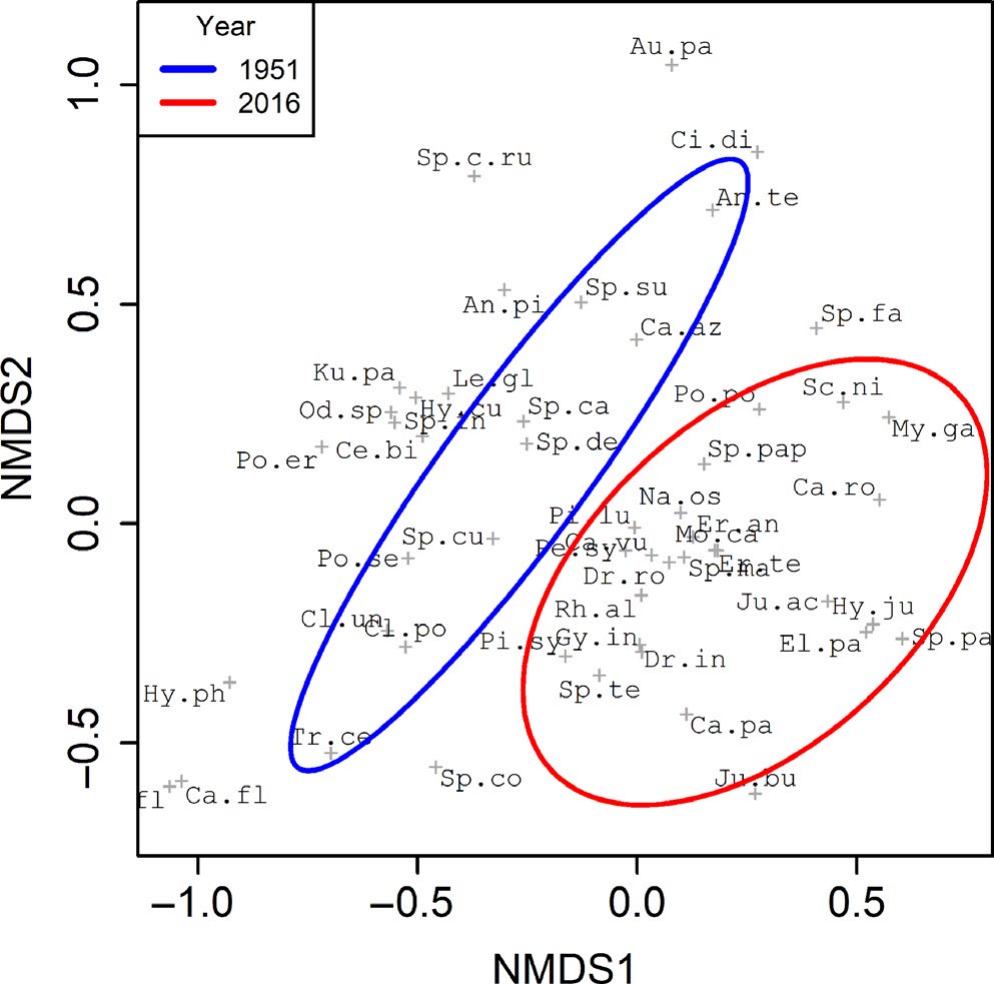
Nonmetric multidimensional scaling (nMDS) of floral community dissimilarity data (*n* = 51, final stress = 0.175) between the 2 years, 1951 and 2016 (represented by blue and red ellipses, respectively). Each “+” represents a single species whose name has been truncated for clarity (see Table S5 for species codes). Ellipses delimit the 95% confidence interval around the centroid for each year

When analyzed by nMDS, changes in vegetation composition were clearly evident, as indicated by a separation between the communities recorded in 1951 and 2016 (Figure 1). Across the site, the species that stayed stable or increased in relative abundance tended to be associated with the bottom right quadrant of the figure, whereas those that declined tended to be associated with the left side of the diagram. The PERMANOVA test revealed a significant effect of survey year on community composition, with 31% of the total variation observed being attributable to the survey date (*F*_1,24_ = 10.628, *r* ^2^ = 0.307, *p* < 0.01). However, when the vegetation plot data were analyzed using MAVIS, little change in NVC community was detected; 11 out of the 13 plots were associated with the same main NVC type at both survey times (Table S6).

### 3.4 Drivers of change

For the landscape survey results, the mean Ellenberg values for L, N, R, Tjan, Tjul, and Prec were significantly different between 1951 and 2016 (all *p* < 0.001) (Table 3). Whereas L and Prec declined over time, N, R, Tjan, and Tjul all increased significantly. When the analyses were repeated including only the 19 species that Newbould originally recorded in 1951, all of the measured Ellenberg traits showed significant differences, with F declining (Table S7). For the vegetation plots, which were characterized by a relatively small sample size but a larger number of constituent species, L was the only Ellenberg value that changed significantly between survey periods. The mean value per plot increased from 7.23 (±0.02) to 7.47 (±0.06) (paired *t* test, *p* = .003), suggesting that there has been a shift toward more light‐demanding species over time.

**TABLE 3:** Results of the Wilcoxon signed‐rank tests comparing the Ellenberg trait values of the landscape survey data between 1951 and 2016

Ellenberg trait	Mean 1951	*SE*	Mean 2016	*SE*	Mean difference	*W*	*p*
L	7.69	0.009	7.48	0.010	−0.210	1,756,268.5	**<0.001**
F	7.72	0.022	7.72	0.022	−0.003	1,232,247	0.059
N	1.57	0.007	1.74	0.011	0.168	1,049,949	**<0.001**
R	2.24	0.017	2.59	0.010	0.353	755,574.5	**<0.001**
Tjan	2.82	0.012	3.11	0.007	0.286	820,039.5	**<0.001**
Tjul	13.39	0.014	13.78	0.005	0.389	555,116	**<0.001**
Prec	1,461.4	3.162	1,330.1	1.547	−131.4	2,142,598.5	**<0.001**

Note

The data analyzed were the mean trait values of all present species in each grid cell. Results in bold indicate a significant difference at *p* < 0.05.

Abbreviations: F, moisture; L, light; N, nitrogen; Prec, precipitation amount; R, pH; Tjan, mean temperature in January; Tjul, mean temperature in July.

A further comparison was made in mean Ellenberg trait values between species that were present in 1951, and those that were absent in 1951 but were present in 2016, using vegetation plot data. Results showed a significant increase in N, R, and Tjul (Wilcoxon rank‐sum tests, *W* = 455.5, 452 and 425.5, respectively, *p* < 0.001 in each case) but a decline in Prec (Wilcoxon rank‐sum tests, *W* = 455.5, *p* < 0.001). This indicates that the species that were newly recorded in 2016 were associated with more fertile soils (N), less acidic substrate (R), higher July temperatures, and a lower level of precipitation, supporting the results of the landscape survey. When Ellenberg numbers of individual species were analyzed, species shown to have declined in abundance over time differed in their mean Ellenberg N (nitrogen) values from those species that showed no decline (paired *t* test, *t* = −2.31, *p = *.025). This suggests that species that declined were less tolerant of high nitrogen availability. None of the other traits were statistically different between survey times.

## 4 DISCUSSION

### 4.1 Vegetation change

On the basis of evidence from peat stratigraphy, mire vegetation has traditionally been viewed as relatively stable in terms of both composition and structure; such stability has been demonstrated over intervals of decades, centuries, and even millennia (Barber, 1981; Gunnarsson et al., 2002; Rydin & Barber, 2001). Our results demonstrate that vegetation composition can change significantly within a single mire over a period of 65 years, as illustrated by the changes in area of occupancy in 58% of species, the decline in species richness in the vegetation plots, and the shift in composition detected in the nMDS and PERMANOVA analyses. In this respect, our results support those obtained in some other studies of mire vegetation conducted over multi‐decadal intervals. For example, a decline in species richness was also recorded by Pellerin et al. (2008) in Québec over a 30‐year period, together with an increase in the percentage cover of trees and species tolerant of shade or drought. At a Swedish site (Skattlösbergs Stormosse) surveyed over a 50‐year interval, Gunnarsson et al. (2000) recorded a decline in species richness, particularly in plots with higher pH. At another Swedish site (Åkhultmyren), Gunnarsson et al. (2002) found no change in species richness over a 40‐year period, but substantial changes in composition were observed; while eight species disappeared, ten new species colonized the site, values that were very similar to those observed here (i.e., 11 new colonists and 11 species lost from the vegetation plots). In addition, 47% of species changed in plot frequency, compared with a value of 58% here. Similarly in the Czech Republic, Hájková et al. (2011) reported significant changes in mire vegetation composition detected using PERMANOVA over a period of up to 60 years, but no change in species richness over time. As noted by Gunnarsson et al. (2002), high turnover and mobility of species is surprising given that all vascular plant species in this type of mire vegetation are perennials, and most are clonal.

Very few previous studies have reported changes in the distribution patterns of individual species at the scale of an entire mire over a multi‐decadal interval; Gunnarsson et al. (2002) provide the only example of which we are aware. Interestingly, some of the changes in species composition reported by Gunnarsson et al. (2002) accorded precisely with those observed here: *H. paludosa* was only observed on the earlier survey date in both studies; *L. inundata* also declined markedly in both investigations. *Rhynchospora fusca* similarly declined in spatial extent in both investigations; while Gunnarsson et al. (2002) identified this as a relatively mobile species, based on the extent to which it entered new plots at the second inventory, in the current study it declined throughout its range and displayed limited ability to colonize new plots (i.e., the species colonized seven new plots since 1951, but disappeared from 79 plots). *Sphagnum cuspidatum* increased its area of occupancy in both studies, as did *P. sylvestris*. In contrast, *S. subnitens* increased in abundance here, whereas it declined in the Swedish investigation.

### 4.2 Processes influencing vegetation

In consideration of their results, Gunnarsson et al. (2002) highlighted a distinction between autogenic and allogenic processes influencing peat‐forming vegetation on their site, the former typically being relatively gradual. Key autogenic processes identified by these authors include the patterns of water flow from the surrounding landscape, changes in the groundwater level and other hydrological changes within the mire, peat accumulation rate, colonization by trees (which can affect growth of other plants by shading), and competition between vascular plants and mosses for light and nutrients. Many of these same processes are likely to have been influential in the current study. In the original survey, Newbould (1960) suggested that the distribution and base status of water flow was the most important factor controlling plant distribution. Specifically, on Cranes Moor two main areas of water flow were identified along the margins, characterized by vegetation dominated by *S. nigricans* or *M. gale* and *M. caerulea,* with areas of *Sphagnum*‐rich vegetation occurring between them. This interpretation was supported by measurements of soil chemistry, water conductivity, and pH, which indicated that distribution of the mire vegetation (as distinct from the marginal wet heath) was related to flushing (Newbould, 1960). Vegetation dominated by *S. papillosum* and *R. alba* was found in an area receiving relatively little water flow from surrounding areas. Additional analysis of pH indicated that the *Sphagnum* communities at Cranes Moor receive some soligenous mineral soil water, based on a pH range of 2.6–4.8 and a modal value of 3.9 (Newbould & Gorham, 1960).

The overall pattern of vegetation recorded here was broadly similar to that described by Newbould (1960), with some expansion of pine woodland particularly on dry heath, and some loss of *Sphagnum* communities to wet heath or *Schoenetum* vegetation (see Figure S2). This implies that the same autogenic processes identified by Newbould (1960) are likely to still be influential. Data obtained from the vegetation plots provide some evidence of localized ecological changes in the mire. For example, the decline in *Eriophorum angustifolium,* which is often associated with cut or eroded peat, and species such as *Sphagnum denticulatum* that are associated with bog pools, might indicate the loss of such pools over time (Rodwell, 1991). As noted by Grant et al. (2014), the part of this site dominated by *Sphagnum* contains a number of regularly spaced pools (up to 5–8 m in length), which are the result of historical peat cutting; since 1951, some of these may have been partially infilled with vegetation. Conversely, the decline of *Calluna vulgaris* and a number of lichen species recorded within the vegetation plots suggests that some areas of relatively dry heath have become inundated over time. The decline in species richness in these plots might be attributable to increasing competition, owing to successional processes; this interpretation is supported by the decline in some light‐demanding species (e.g., *N. ossifragum*) and an increase in others (e.g., *S. palustre*) that are relatively shade tolerant.

At the scale of the entire site, the most striking changes in vegetation composition were the spread of bracken (*P. aquilinum*) and Scots pine (*P. sylvestris*) saplings, and the increase in two *Sphagnum* species, *S*. *cuspidatum* and *S. subnitens*. The sensitivity of bracken to waterlogging is well established (Marrs & Watt, 2006). Similarly, Scots pine is also sensitive to a high water table, especially as a seedling (Pearson et al., 2011), although it can also demonstrate a degree of physiological tolerance of waterlogging (Pearson et al., 2013). Together, these observations suggest some surface drying of the site, perhaps reflecting a lowering of the water table. Conversely, the spread of two *Sphagnum* species suggest an opposing trend: some parts of the site appear to have become wetter. Interestingly, these two *Sphagnum* species are characterized by different microsite preferences; whereas *S. cuspidatum* is typically a species of acidic pools, *S. subnitens* is generally associated with richer fen vegetation (Gunnarsson et al., 2002). Taken together, these results highlight the spatial heterogeneity in how the vegetation of the site has changed over time, in response to fluctuations in both rainfall and groundwater flow, and well as the surface topography and hydrology of the mire.

Allogenic processes also appear to have had a major influence on vegetation change in Cranes Moor. Analysis of the Ellenberg trait values for the landscape survey results (Table 3) indicates a number of shifts in the functional composition of the vegetation over time, providing evidence of increased relative abundance of species that are relatively tolerant of shade and drought, and those that are associated with higher temperatures and higher nitrogen availability. These findings are consistent with those of other studies of mire vegetation conducted over multi‐decadal intervals. For example in Sweden, Gunnarsson et al. (2002) similarly observed an increase in species associated with higher nitrogen availability, higher shade tolerance, and lower moisture availability, based on analysis of Ellenberg trait values. Based on this evidence, these authors concluded that many of the vegetation changes they observed were allogenic in origin and were caused by the combined effects of increased surface dryness, acidification, and increased nitrogen supply. In Québec, Pellerin et al. (2008) also found an increase in the abundance of species tolerant of shade and drought, whereas in the Czech Republic, observed vegetation changes were attributed at least partly to increased nutrient concentration resulting from atmospheric deposition (Hájková et al., 2011).

### 4.3 Caveats

Analysis of long‐term change in plant communities is typically associated with a degree of uncertainty, owing to potential errors in estimation of species losses and gains, which can arise from imprecise location of the original plots or variation in survey effort (Bennie et al., 2006). The current study is no exception in this respect (Appendix S1). A further factor is the conditions in the year in which the study was undertaken, which applies to the original study as much to the resurvey; Newbould (1953) notes that 1951 was a particularly wet year. As a consequence, the results should be viewed with caution. One key unknown is the impact of previous disturbance of the site. Newbould (1960) mentions that the site may have previously been disturbed by burning, turf‐cutting, and military maneuvers. Similarly, the area of surrounding heathland is subjected to management interventions that include cutting of pine and occasional burning (Figure S6). Grant et al. (2014) also mention that the central area of the site contains a number of large pools (up to 5 × 8 m), which may be the result of past peat cutting. The incidence and potential impact of such disturbances are unknown, but they may have influenced some of the results observed, including the spatial heterogeneity in the responses of different species. The inconsistent results obtained in the Ellenberg L value between the landscape survey and vegetation plots may similarly be attributable to spatial heterogeneity in disturbance history and its impact on competitive interactions between different species, although the relatively low statistical power of the vegetation plots might also have been a factor in this case.

### 4.4 Management implications

Given these caveats, the evidence presented here tentatively suggests that this mire system has changed significantly in ecological condition over the past 65 years, which has implications for its conservation and management. In this context, the main issues of concern are the overall decline in species richness, the shift in species composition, and the decline and/or loss of some distinctive elements of the vegetation, such as *R. fusca*, *L. inundata,* and *H. paludosa*. If these trends are being driven by increased surface dryness and increased atmospheric nitrogen supply, as these results suggest, then appropriate management responses to these threats will need to be identified.

At the scale of the entire UK, nutrient enrichment resulting from atmospheric nitrogen deposition has been identified as a major driver of vegetation change in recent decades (Haines‐Young et al., 2003; Payne et al., 2017). Bog vegetation is known to be particularly vulnerable to nitrogen deposition, and in the UK, virtually all bogs are now believed to be at or above the critical load for this element (JNCC, 2011). Negative impacts of nitrogen deposition on vegetation have been widely observed throughout Europe (Dise et al., 2011), as well as other parts of the world, although the magnitude of these effects has rarely been documented in detail (Payne et al., 2017). When taken together with results of other long‐term studies of mire vegetation, the analyses presented here provide clear evidence of significant changes in vegetation composition attributable to this factor. An additional potential driver is anthropogenic climate change, which may account for the shift toward species with higher drought and temperature tolerances observed here, given that mean temperatures and the incidence of drought have increased at this location in recent decades (Martin et al., 2015).

Concerns about the potential impact of climate change on mire vegetation are well established (Page & Baird, 2016); for example, climate envelope modeling indicates that in the UK, the distribution patterns of rain‐fed mires such as blanket bog are highly sensitive to climate (Gallego‐Sala et al., 2010). Analysis of 56 peat bog ecosystems across Europe found that temperature and precipitation were the most important drivers of vegetation composition, followed by atmospheric nitrogen deposition (Robroek et al., 2017), a finding consistent with the results of this study. These results are also supported by analysis of vegetation change over a 70‐year interval in the county of Dorset, which neighbors the study site examined here. Analysis of changes in functional traits in heathland (Diaz et al., 2013), calcareous grassland (Newton et al., 2012), and woodland (Keith et al., 2009, 2011) consistently indicated that nitrogen eutrophication was the principal driver of changes in community composition, with climate change also implicated in heathland and grassland.

It is recognized that for sensitive habitats such as lowland mires located near to agricultural sources of nitrogen, spatially targeted measures to reduce local emissions can potentially enable local reductions in nitrogen deposition to be achieved (Plantlife, 2017). However, the site investigated here is located remotely from agricultural land and other nitrogen sources, at least compared to other sites in lowland England. Regional or national‐scale reduction strategies, such as that presented by Defra (2018), may therefore be required. Even if a reduction in nitrogen deposition was achieved, recovery of the plant community could be very slow. While some soil variables, such as nitrate and ammonium concentrations, can respond relatively rapidly to reductions in nitrogen inputs, variables such as the species composition of vegetation or of below‐ground communities typically recover at a much lower rate (Stevens, 2016). Complete recovery may require additional interventions to be performed, such as physical removal of accumulated nitrogen or reintroduction of species that have been lost from the site (Plantlife, 2017; Stevens, 2016; Stevens et al., 2013).

Mitigation of climate change similarly requires coordinated action to reduce greenhouse gas emissions at national and international scales. However, actions aimed at stopping or reversing the deterioration of individual mire ecosystems caused by other threats can itself be considered as a contribution to climate change mitigation (Essl et al., 2012). A wide range of management interventions have been implemented on different peatlands with the aim of reducing desiccation, such as blocking ditches to raise the water table; building bunds to retain water and distribute it more evenly; and provision of open‐water reservoirs to increase lateral seepage (Grzybowski & Glińska‐Lewczuk, 2020). Although evidence regarding the effectiveness of these interventions is limited in extent and variable in quality, rewetting by raising the water table has consistently been found to have beneficial effects on peatland ecosystems, such as increasing the cover of characteristic plant species (Taylor et al., 2019). Such interventions are therefore recommended for the site examined here, and for other lowland mires affected by similar environmental pressures.

## CONFLICT OF INTEREST

None declared.

## AUTHOR CONTRIBUTIONS

**Alexander T. Lovegrove:** Conceptualization (equal); formal analysis (lead); investigation (lead); methodology (equal); writing – original draft (supporting). **Adrian C. Newton:** Conceptualization (equal); formal analysis (supporting); funding acquisition (lead); investigation (supporting); methodology (equal); project administration (lead); supervision (lead); writing – original draft (lead); writing – review and editing (lead). **Paul M. Evans:** Data curation (lead); formal analysis (lead); investigation (equal); visualization (lead); writing – original draft (supporting). **Anita Diaz:** Investigation (supporting); writing – review and editing (supporting). **Arthur C. Newton:** Investigation (supporting); visualization (supporting). **Lynn Davy:** Investigation (supporting); supervision (supporting); writing – review and editing (supporting). **Palmer J. Newbould:** Conceptualization (equal); methodology (equal).

## Supporting information

Appendix S1

## Data Availability

The data are made available on Figshare https://doi.org/10.6084/m9.figshare.13102541.
