# Supplementary material for: Changes in vegetation structure and composition of a lowland mire over a sixty‐five‐year interval
Source: Ecol Evol. 2020 Nov 12;10(24):13913–25. doi: 10.1002/ece3.6984 (PMC7771134; doi:10.1002/ece3.6984)
Supplement: Supplementary file 1 — Appendix S1 [file ECE3-10-13913-s001.docx]

***Electronic Supplementary Material***

**Changes in vegetation structure and composition of a lowland mire over a sixty-five year interval**

Alexander T. Lovegrove^1^, Adrian C. Newton^1*^, Paul M. Evans^1^, Anita Diaz^1^, Arthur C. Newton^2^, Lynn Davy^3^ and Palmer J. Newbould^4^

^1^Centre for Ecology, Environment and Sustainability, Faculty of Science and Engineering, Bournemouth University, BH12 5BB, UK.

^2^College of Life and Environmental Sciences, University of Exeter, Penryn, Cornwall, TR10 9EZ, UK

^3^20 Highland Road, Wimborne, Dorset, BH21 2QN, UK.

^4±^26 Morestall Drive, Cirencester, Gloucestershire GL7 1TF, UK.

^±^ Deceased

**Appendix S1** Supplementary information on methods

**Table S1**. Change in species on the Cranes Moor plots between 1951 and 2016, including new species records.

**Table S2**. List of species that were absent from the vegetation plots in one of the survey years.

**Table S3.** Species that did not show statistically significant declines (i.e. class as increasing or stable) in 20 x 20 m plots or occupancy over the whole of Cranes Moor, and their associated Ellenberg values.

**Table S4.** Species that showed statistically significant declines in 20 x 20 m plots or occupancy over the whole of Cranes Moor, and their associated Ellenberg values.

**Table S5.** Abbreviated species names.

**Table S6.** NVC communities of the vegetation plot data, for both 1951 and 2016.

**Table S7.** Wilcoxon signed-rank tests comparing the Ellenberg trait values of the landscape survey between 1951 and 2016, using only the 19 species featured in Newbould’s original landscape survey.

**Table S8.** Species mapped on the Cranes Moor survey in 1951 and in the present survey (2016).

**Figure S1.**  Map of Cranes Moor.

**Figure S2**. Vegetation map of Cranes Moor in 1951.

**Figure S3**. Map showing the change in abundance changes in species which did not differ significantly between 1951 and 2016.

**Figure S4**. Map showing the abundance of species recorded and mapped in 2016.

**Figure S5**. Maps showing abundance changes in species which did not differ significantly between 1951 and 2016.

**Figure S6**. Photographs of Cranes Moor in 1951 and 2016.

**Appendix S1 Supplementary information on methods**

It is important that the current results be viewed in the context of the limitations of this type of study. A first limitation is the fact that surveys were only completed on two dates; a fuller analysis of community dynamics would require a number of repeat surveys to be conducted over time. A second potential problem is that of errors in estimation of species losses and gains (pseudo-turnover, Fischer & Stöcklin 1997) owing to imprecise location of the original plots, or differences in the sampling intensity or taxonomic expertise of the surveyors involved at different times. While the original plots were carefully mapped and relatively easy to relocate, it is possible that there were errors associated with the precise relocation of the survey plots; furthermore, implementation of the sampling approach may have differed between the two surveys. This could potentially lead to errors in estimates of species richness, which might either be reduced or inflated. In the current investigation, any taxonomic changes that have occurred since the original survey were addressed where necessary by converting original names to those employed by recent floras (i.e. Stace 2010). Neverthless, there may have been misidentification of individual specimens, which will have been more likely in critical genera such as *Sphagnum* and *Carex*. It is also possible that survey effort differed between the two surveys, but this is difficult to estimate with precision. These potential sources of uncertainty should be considered when interpreting the results.

**References**

Fischer, M. and Stöcklin, J. (1997). Local extinctions of plants in remnants of extensively used calcareous grasslands 1950–1985. *Conservation Biology*, *11*, 727-737.

Stace, C. (2010). *New Flora of the British Isles*, 3rd Edition. Cambridge University Press, Cambridge.

| **Table S1**. Change in species composition in the Cranes Moor vegetation plots between 1951 and 2016, including new species records. | | |  |
| --- | --- | --- | --- |
| Present in 1951, but not recorded in 2016 | Present in 2016, but not recorded in 1951 |  | |
| *Aulacomnium palustre* | *Betula pendula* |  | |
| *Campylopus flexuosus** | *Carex viridula* | |  |
| *Diplophyllum albicans* | *Dactylorhizus maculata* | |  |
| *Hammarbya paludosa Hypogymnia physodes*  *Kurzia pauciflora*  *Lycopodiella inundata**  *Potentilla erecta**  *Quercus robur**  *Ulex minor**  *Utricularia minor** | *Dicranium scoparium*  *Eleocharis palustris*  *Eleocharis quinquefolia*  *Juncus buffonis*  *Juncus bulbosus*  *Juncus compressus*  *Menyanthes trifoliata*  *Sphagnum palustre* | |  |
|  |  | |  |
| * These species were encountered during the mapping survey in 2016 at different locations, so they are not completely absent from Cranes Moor. | | |  |

**Table S2**. List of species that were absent from the vegetation plots one of the survey years.

| Missing species | Year absent  from vegetation  plots |
| --- | --- |
| *Aulacomnium palustre* | Absent in 2016 |
| *Campylopus flexuosus* | Absent in 2016 |
| *Diplophyllum albicans* | Absent in 2016 |
| *Kurzia pauciflora* | Absent in 2016 |
| *Lycopodiella inundata* | Absent in 2016 |
| *Potentilla erecta* | Absent in 2016 |
| *Quercus robur* | Absent in 2016 |
| *Sphagnum capillifolium* subsp*. rubellum* | Absent in 2016 |
| *Ulex minor* | Absent in 2016 |
| *Utricularia minor* | Absent in 2016 |
| *Betula pendula* | Absent in 1951 |
| *Carex viridula* subsp. *oedocarpa* | Absent in 1951 |
| *Dactylorhiza maculata* | Absent in 1951 |
| *Dicranum scoparium* | Absent in 1951 |
| *Eleocharis palustris* | Absent in 1951 |
| *Eleocharis quinqueflora* | Absent in 1951 |
| *Hypnum jutlandicum* | Absent in 1951 |
| *Juncus bufonius* | Absent in 1951 |
| *Juncus bulbosus* | Absent in 1951 |
| *Juncus compressus* | Absent in 1951 |
| *Menyanthes trifoliata* | Absent in 1951 |
| *Sphagnum palustre* | Absent in 1951 |

**Table S3.** Species that did not show statistically significant declines (i.e. classed as increasing or stable) in vegetation plots or in occupancy over the whole of Cranes Moor, and their associated Ellenberg values. The V/L column indicates whether the species did not show significant declines in the vegetation plots (V), the landscape survey (L) or both (V/L). Abbreviations: N, nitrogen, L, light, R, pH, F, moisture, Tjan, mean temperature in January, Tjul, mean temperature in July, and Prec, precipitation amount.

| Species name | L | F | N | R | Tjan | Tjul | Prec | V/L |
| --- | --- | --- | --- | --- | --- | --- | --- | --- |
| *Anagallis tenella* | 8 | 8 | 3 | 5 | 4 | 14.6 | 1139 | V |
| *Aulacomnium palustre* | 7 | 8 | 2 | 3 | 3.1 | 14 | 1263 | V |
| *Betula pendula* | 7 | 5 | 4 | 4 | 3.3 | 14.6 | 1073 | V |
| *Campylopus flexuosus* | 6 | 6 | 1 | 2 | 3.3 | 14.2 | 1255 | V |
| *Carex panicea* | 8 | 8 | 2 | 4 | 3.5 | 14.3 | 1139 | V |
| *Carex rostrata* | 8 | 10 | 2 | 4 | 3.3 | 14 | 1193 | V |
| *Carex viridula* subsp*. oedocarpa* | 8 | 8 | 2 | 4 | 3.4 | 14.2 | 1194 | V |
| *Cirsium dissectum* | 8 | 8 | 2 | 4 | 4 | 15 | 1043 | V |
| *Dactylorhiza maculata* | 7 | 7 | 2 | 3 | 3.4 | 14.1 | 1221 | V |
| *Dicranum scoparium* | 6 | 5 | 2 | 3 | 3.4 | 14.4 | 1150 | V |
| *Diplophyllum albicans* | 5 | 6 | 1 | 2 | 3.3 | 14 | 1286 | V |
| *Drosera intermedia* | 8 | 9 | 1 | 2 | 3.7 | 14.1 | 1337 | V |
| *Drosera rotundifolia* | 8 | 9 | 1 | 2 | 3.3 | 13.9 | 1269 | V |
| *Eleocharis palustris* | 8 | 10 | 4 | 6 | 3.5 | 14.5 | 1098 | V |
| *Eleocharis quinqueflora* | 9 | 9 | 2 | 7 | 3.1 | 13.5 | 1323 | V |
| *Erica cinerea* | 7 | 5 | 2 | 2 | 3.5 | 14 | 1226 | L |
| *Gymnocolea inflata* | 7 | 7 | 1 | 1 | 3.1 | 14.1 | 1266 | V |
| *Hypnum jutlandicum* | 6 | 5 | 2 | 2 | 3.4 | 14.2 | 1206 | V |
| *Juncus acutiflorus* | 8 | 8 | 2 | 4 | 3.5 | 14.5 | 1115 | V |
| *Juncus bufonius* | 7 | 7 | 5 | 6 | 3.6 | 14.6 | 1102 | V |
| *Juncus bulbosus* | 7 | 10 | 2 | 4 | 3.5 | 14.2 | 1180 | V |
| *Juncus compressus* | 8 | 8 | 5 | 7 | 3.6 | 15.9 | 746 | V |
| *Leucobryum glaucum* | 5 | 6 | 2 | 2 | 3.2 | 14 | 1333 | V |
| *Lycopodiella inundata* | 9 | 9 | 1 | 2 | 3.5 | 14.9 | 1122 | V |
| *Menyanthes trifoliata* | 8 | 10 | 3 | 4 | 3.4 | 14.1 | 1201 | V/L |
| *Molinia caerulea* | 7 | 8 | 2 | 3 | 3.5 | 14.2 | 1179 | V |
| *Myrica gale* | 8 | 9 | 2 | 3 | 3.4 | 13.8 | 1353 | V/L |
| *Pedicularis sylvatica* | 8 | 8 | 2 | 3 | 3.4 | 14.1 | 1202 | V |
| *Pinguicula lusitanica* | 8 | 8 | 2 | 4 | 3.9 | 13.7 | 1475 | V |
| *Pinus sylvestris* | 7 | 6 | 2 | 2 | 0.8 | 11.7 | 1930 | V/L |
| *Potamogeton polygonifolius* | 8 | 10 | 2 | 4 | 3.3 | 13.9 | 1254 | V |
| *Potentilla erecta* | 7 | 7 | 2 | 3 | 3.5 | 14.4 | 1118 | V |
| *Pteridium aquilinum* | 6 | 5 | 3 | 3 | 3.5 | 14.5 | 1109 | L |
| *Quercus robur* | 7 | 5 | 4 | 5 | 3.5 | 14.7 | 1049 | V |
| *Rhynchospora alba* | 8 | 9 | 1 | 2 | 3.6 | 14 | 1400 | V |
| *Schoenus nigricans* | 8 | 8 | 2 | 7 | 3.9 | 13.9 | 1261 | V/L |
| *Sphagnum capillifolium* | 7 | 7 | 1 | 2 | 3 | 13.6 | 1387 | V |
| *Sphagnum capillifolium* subsp*. rubellum* | 7 | 7 | 1 | 2 | 3.8 | 14.9 | 1217 | V |
| *Sphagnum compactum* | 8 | 8 | 1 | 1 | 3 | 13.4 | 1466 | V |
| *Sphagnum cuspidatum* | 8 | 10 | 2 | 1 | 3.1 | 13.7 | 1361 | V/L |
| *Sphagnum fallax* | 7 | 9 | 3 | 2 | 3.1 | 14.3 | 1306 | V |
| *Sphagnum magellanicum* | 8 | 8 | 1 | 1 | 2.9 | 13.4 | 1418 | V/L |
| *Sphagnum palustre* | 7 | 8 | 2 | 3 | 3.1 | 13.9 | 1292 | V |
| *Sphagnum papillosum* | 8 | 8 | 1 | 1 | 3.1 | 13.6 | 1393 | V/L |
| *Sphagnum subnitens* | 7 | 8 | 2 | 3 | 3.2 | 13.8 | 1332 | V/L |
| *Sphagnum tenellum* | 8 | 8 | 1 | 1 | 3.2 | 13.6 | 1428 | V |
| *Trichophorum cespitosum* | 8 | 8 | 1 | 2 | 3.2 | 13.7 | 1312 | V |
| *Ulex europaeus* | 7 | 5 | 3 | 5 | 3.6 | 14.6 | 1090 | L |
| *Ulex minor* | 8 | 6 | 2 | 1 | 3.9 | 16.4 | 745 | V |
| *Utricularia minor* | 8 | 12 | 2 | 4 | 3.6 | 13.9 | 1321 | V |
| Mean +/- (SE) | 7.42 (0.122) | 7.72 (0.232) | 2.06 (0.141) | 3.14 (0.23) | 3.36 (0.064) | 14.152 (0.093) | 1237.56 (25.276) |  |

**Table S4.** Species that showed statistically significant declines in 20 x 20 m plots or occupancy over the whole of Cranes Moor, and their associated Ellenberg values. The V/L column indicates whether the species showed significant declines in the vegetation plots (V), the landscape survey (L) or both (V/L). Abbreviations: N, nitrogen, L, light, R, pH, F, moisture, Tjan, mean temperature in January, Tjul, mean temperature in July, and Prec, precipitation amount.

| **Species name** | **L** | **F** | **N** | **R** | **Tjan** | **Tjul** | **Prec** | **V/L** |
| --- | --- | --- | --- | --- | --- | --- | --- | --- |
| *Aneura pinguis* | 8 | 9 | 2 | 6 | 3.3 | 14.2 | 1220 | V |
| *Calluna vulgaris* | 7 | 6 | 2 | 2 | 3.5 | 14.3 | 1157 | V |
| *Calypogeia azurea* | 3 | 7 | 1 | 3 | 1.9 | 12.6 | 1658 | V |
| *Carex rostrata* | 8 | 10 | 2 | 4 | 3.3 | 14 | 1193 | L |
| *Cephalozia bicuspidata* | 4 | 7 | 2 | 2 | 3.3 | 14.2 | 1220 | V |
| *Cirsium dissectum* | 8 | 8 | 2 | 4 | 4 | 15 | 1043 | L |
| *Erica tetralix* | 8 | 8 | 1 | 2 | 3.4 | 14 | 1230 | V |
| *Eriophorum angustifolium* | 8 | 9 | 1 | 4 | 3.4 | 14.1 | 1194 | V |
| *Hypnum cupressiforme* | 6 | 4 | 4 | 4 | 3.4 | 14.6 | 1098 | V |
| *Kurzia pauciflora* | 7 | 9 | 1 | 1 | 3.3 | 13.8 | 1404 | V |
| *Lycopodiella inundata* | 9 | 9 | 1 | 2 | 3.5 | 14.9 | 1122 | L |
| *Narthecium ossifragum* | 8 | 9 | 1 | 2 | 3.3 | 13.8 | 1296 | V |
| *Odontoschisma sphagni* | 8 | 8 | 1 | 1 | 3.3 | 13.6 | 1436 | V |
| *Polygala serpyllifolia* | 8 | 7 | 2 | 2 | 3.4 | 14.1 | 1200 | V |
| *Rhynchospora alba* | 8 | 9 | 1 | 2 | 3.6 | 14 | 1400 | L |
| *Rhynchospora fusca* | 9 | 9 | 1 | 3 | 4.4 | 14.7 | 1205 | L |
| *Sphagnum compactum* | 8 | 8 | 1 | 1 | 3 | 13.4 | 1466 | L |
| *Sphagnum denticulatum* | 7 | 9 | 2 | 2 | 3.2 | 13.9 | 1353 | V |
| *Sphagnum inundatum* | 7 | 9 | 2 | 3 | 3.1 | 13.7 | 1426 | V |
| *Trichophorum cespitosum* | 8 | 8 | 1 | 2 | 3.2 | 13.7 | 1312 | L |
| **Mean +/- (SE)** | 7.35 (0.335) | 8.1 (0.307) | 1.55 (0.17) | 2.6 (0.285) | 3.34 (0.102) | 14.03 (0.121) | 1281.65 (33.464) |  |

**Table S5.** Abbreviated species names.

| Species name | Abbreviation |
| --- | --- |
| *Anagallis tenella* | An.te |
| *Calluna vulgaris* | Ca.vu |
| *Carex panicea* | Ca.pa |
| *Carex rostrata* | Ca.ro |
| *Cirsium dissectum* | Ci.di |
| *Drosera intermedia* | Dr.in |
| *Drosera rotundifolia* | Dr.ro |
| *Erica tetralix* | Er.te |
| *Eriophorum angustifolium* | Er.an |
| *Juncus acutiflorus* | Ju.ac |
| *Molinia caerulea* | Mo.ca |
| *Myrica gale* | My.ga |
| *Narthecium ossifragum* | Na.os |
| *Pedicularis sylvatica* | Pe.sy |
| *Pinguicula lusitanica* | Pi.lu |
| *Pinus sylvestris* | Pi.sy |
| *Polygala serpyllifolia* | Po.se |
| *Potamogeton polygonifolius* | Po.po |
| *Potentilla erecta* | Po.er |
| *Rhynchospora alba* | Rh.al |
| *Schoenus nigricans* | Sc.ni |
| *Trichophorum cespitosum* | Tr.ce |
| *Juncus bufonius* | Ju.bu |
| *Eleocharis palustris* | El.pa |
| *Sphagnum compactum* | Sp.co |
| *Sphagnum cuspidatum* | Sp.cu |
| *Sphagnum magellanicum* | Sp.ma |
| *Sphagnum capillifolium* | Sp.ca |
| *Sphagnum capillifolium subsp rubellum* | Sp.c.ru |
| *Sphagnum subnitens* | Sp.su |
| *Sphagnum fallax* | Sp.fa |
| *Sphagnum papillosum* | Sp.pap |
| *Sphagnum denticulatum* | Sp.de |
| *Sphagnum tenellum* | Sp.te |
| *Sphagnum inundatum* | Sp.in |
| *Sphagnum palustre* | Sp.pal |
| *Aulacomnium palustre* | Au.pa |
| *Calypogeia azurea* | Ca.az |
| *Campylopus flexuosus* | Ca.fl |
| *Cephalozia bicuspidata* | Ce.bi |
| *Aneura pinguis* | An.pi |
| *Gymnocolea inflata* | Gy.in |
| *Hypnum cupressiforme* | Hy.cu |
| *Hypnum jutlandicum* | Hy.ju |
| *Kurzia pauciflora* | Ku.pa |
| *Leucobryum glaucum* | Le.gl |
| *Odontoschisma sphagni* | Od.sp |
| *Cladonia floerkeana* | Cl.fl |
| *Cladonia uncialis* | Cl.un |
| *Cladonia portentosa* | Cl.po |
| *Hypogymnia physodes* | Hy.ph |
|  |  |

**Table S6.** NVC communities of the vegetation plot data, for both 1951 and 2016, as determined using MAVIS (Smart et al., 2016; see main text). The communities with the highest coefficients are presented. Values of Shannon’s Index of Diversity are also presented for each plot. Mean values of the Index declined between the two survey intervals (*P* < 0.001, paired *t*-test).

| **Vegetation plot number** | **NVC community (MATCH coefficient)** | | **Shannon’s Index of Diversity** | |
| --- | --- | --- | --- | --- |
|  | **1951** | **2016** | **1951** | **2016** |
| I | M16a (59.79%) | M16a (61.88%) | 1.18 | 1.08 |
| II | M16c (63.62%) | M21 (66.91%) | 1.27 | 1.06 |
| III | M16a (64.41%) | M16 (57.58%) | 1.27 | 1.11 |
| IV | M21 (65.65%) | M21 (63.72%) | 1.19 | 1.07 |
| V | M21 (60.65%) | M1 (37.41%) | 1.23 | 1.17 |
| VI | M21 (69.01%) | M21 (58.94%) | 1.25 | 1.10 |
| VII | M21 (52.24%) | M21 (54.01%) | 1.25 | 1.15 |
| VIII | M21 (65.15%) | M21 (63.37%) | 1.20 | 1.06 |
| IX | M21b (61.78%) | M21 (56.31%) | 1.12 | 1.12 |
| X | M21 (60.15%) | M21 (66.99%) | 1.27 | 0.99 |
| XI | M21 (56.67%) | M21 (61.59%) | 1.28 | 1.10 |
| XII | M21 (67.70%) | M25a (51.55%) | 1.30 | 0.87 |
| XIII | M21 (75.00%) | M21 (63.37%) | 1.26 | 1.03 |

**Table S7.** Results for the Wilcoxon signed-rank tests comparing the Ellenberg trait values of the landscape survey between 1951 and 2016, using only the 19 species featured in Newbould’s original landscape survey. The data analysed were the mean trait values of all present species in each grid cell. Results in bold indicate a significant difference at P < 0.05. Abbreviations: N, nitrogen, L, light, R, pH, F, moisture, Tjan, mean temperature in January, Tjul, mean temperature in July, and Prec, precipitation amount.

| Ellenberg trait | Mean 1951 | SE | Mean 2016 | SE | Mean  Difference | *W* | *p* |
| --- | --- | --- | --- | --- | --- | --- | --- |
| L | 7.70 | 0.007 | 7.56 | 0.011 | -0.138 | 681158 | **< 0.001** |
| F | 7.73 | 0.021 | 7.52 | 0.027 | -0.207 | 705763.5 | **< 0.001** |
| N | 1.57 | 0.007 | 1.71 | 0.009 | 0.137 | 255152.5 | **< 0.001** |
| R | 2.24 | 0.017 | 2.29 | 0.017 | 0.05 | 467209 | **0.001** |
| Tjan | 2.83 | 0.012 | 2.75 | 0.013 | -0.074 | 707803.5 | **< 0.001** |
| Tjul | 13.40 | 0.011 | 13.35 | 0.011 | -0.053 | 697360.5 | **< 0.001** |
| Prec | 1461.91 | 3.017 | 1468.38 | 3.167 | 6.467 | 561824 | **0.002** |

**Table S8.** Species mapped on the Cranes Moor survey in 1951 and in the present survey (2016).

|  | |
| --- | --- |
| 1951 and 2016 surveys | 2016 survey only |
| *Carex rostrata* | *Betula* sp.* |
| *Cirsium dissectum* | *Calluna vulgaris* |
| *Erica cinerea* | *Campylopus introflexus* |
| *Lycopodiella inundata*** | *Cladonia* sp. |
| *Menyanthes trifoliata* | *Dactylorhiza* sp. |
| *Myrica gale* | *Sphagnum denticulatum* agg. |
| *Pinus sylvestris** | *Eleocharis* sp. |
| *Pteridium aquilinum* | *Equisetum fluviatile* |
| *Rhynchospora alba* | *Erica tetralix* |
| *Rhynchospora fusca* | *Ilex aquifolium* |
| *Schoenus nigricans* | *Juncus acutiflorus* |
| *Sphagnum compactum* | *Molinia caerulea* |
| *Sphagnum cuspidatum* | *Narthecium ossifragum* |
| *Sphagnum magellanicum* | *Potomageton polygonifolius* |
| *Sphagnum papillosum* |  |
| *Sphagnum subnitens*** |  |
| *Trichophorum germanicum*** |  |
| *Ulex europaeus*  *Hammarbya paludosa**** |  |
| * Recorded separately for seedlings, saplings, and trees | |
| ** Recorded under a different name in Newbould (1960); *Lycopodiella inundata* = *Lycopodium inundatum, Sphagnum subnitens* = *Sphagnum plumulosum* and *Trichophorum germanicum* = *Scirpus cespitosus.*  ***** Species not detected in 2016 survey | |


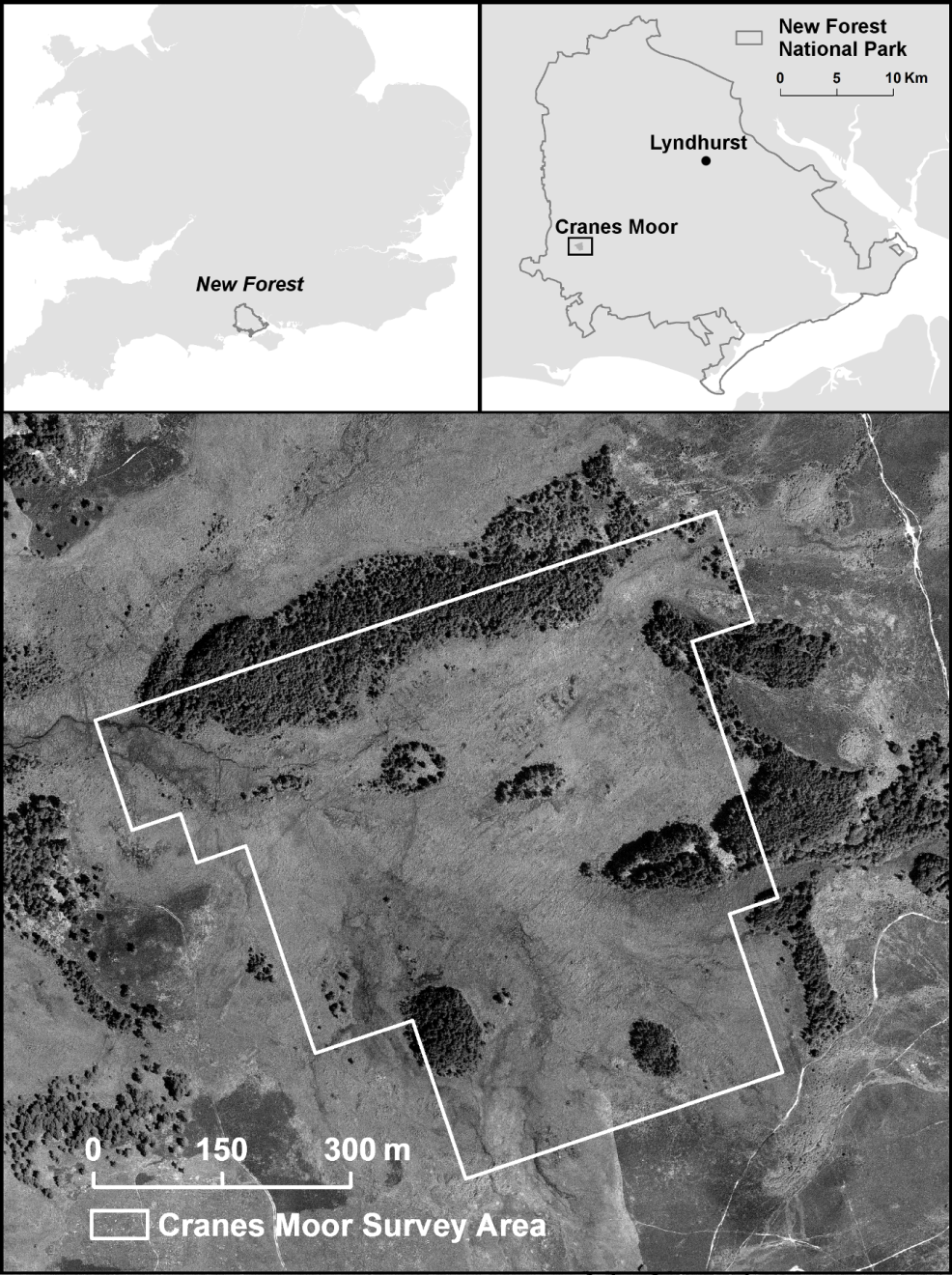


**Figure S1**. Map of Cranes Moor, showing the location of the New Forest National Park within the UK (upper left), the location of the study area within the New Forest (upper right) and the area surveyed by Newbould (1960) and this study (below). The bottom of the photograph is the southern edge. Some of the trees visible in the photo have since been removed from the site. Aerial photograph from Infoterra Ltd, 2000.


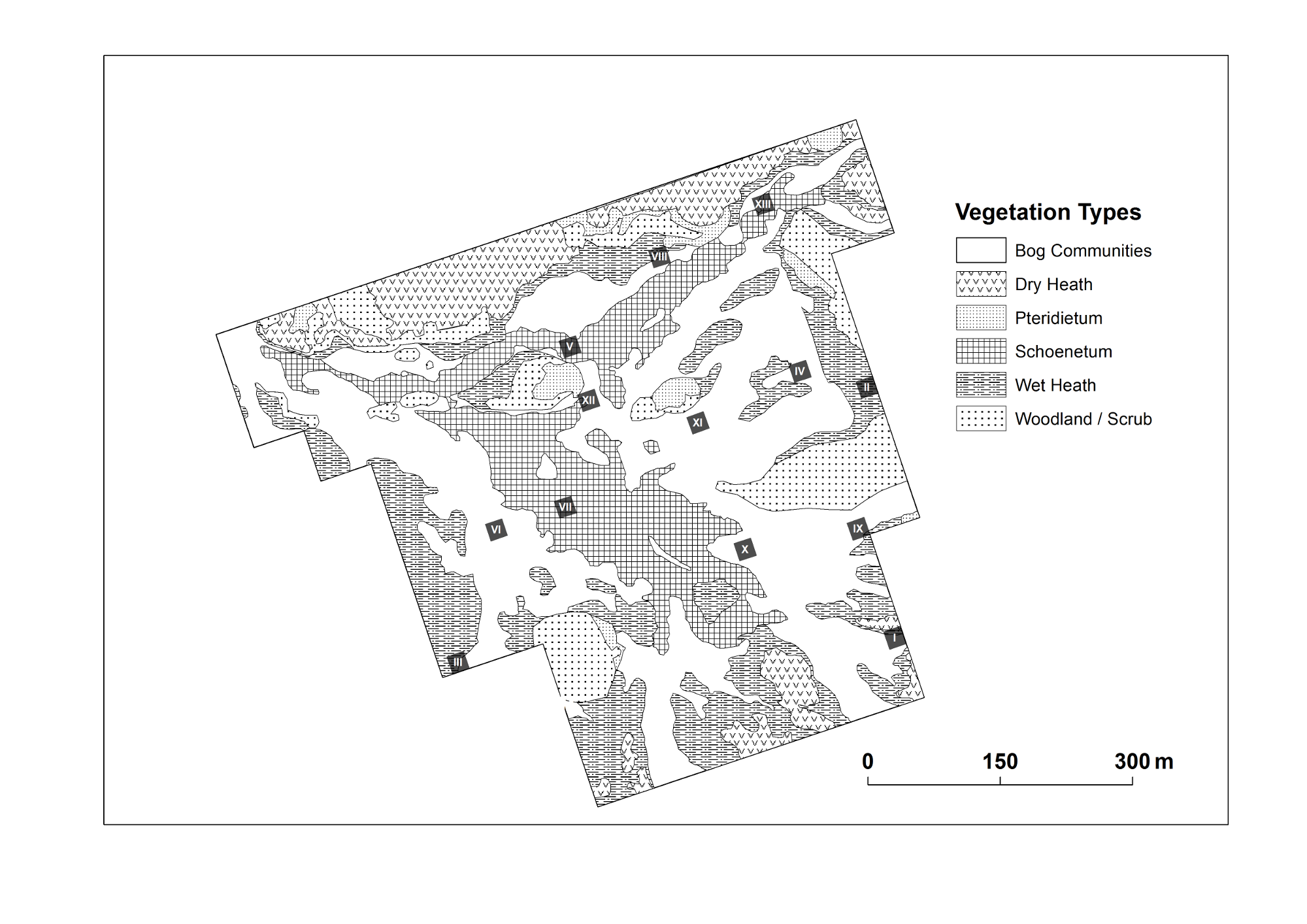


**Figure S2**. Map showing different vegetation types on Cranes Moor, based on Newbould’s (1960) classification and mapped using a combination of aerial photography and distribution data from the present study. The Latin numerals indicate the position of the vegetation plots.


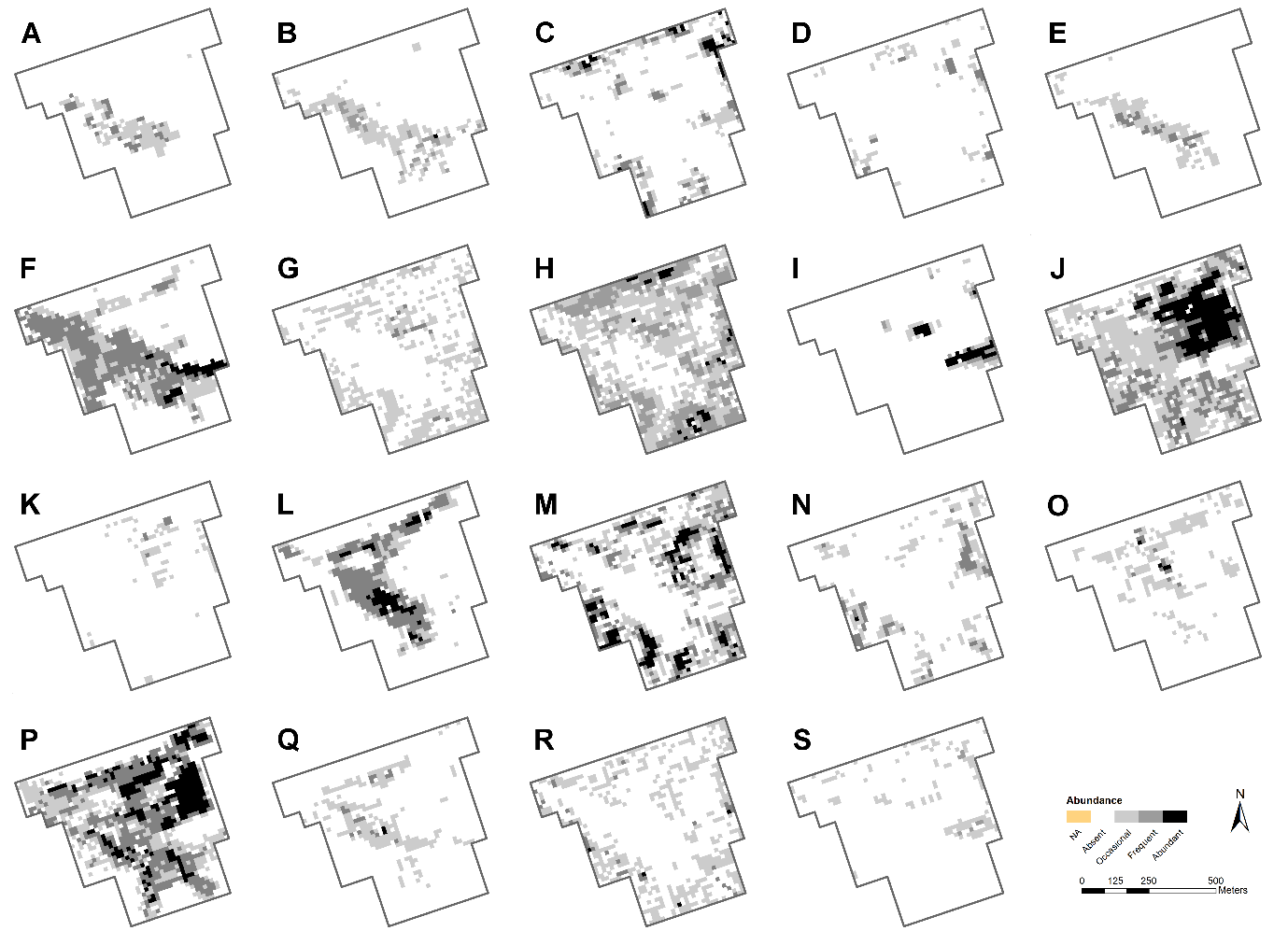


**Figure S3**. Map showing the distribution of species surveyed by Newbould in 1951. A – *Carex rostrata*, B – *Cirsium dissectum*, C – *Erica cinerea*, D – *Lycopodiella inundata*, E – *Menyanthes trifoliata*, F – *Myrica gale*, G – *Pinus sylvestris* (sapling), H – *Pinus sylvestris* (seedling), I – *Pteridium aquilinum*, J – *Rhynchospora alba*, K – *Rhynchospora fusca*, L – *Schoenus nigricans*, M – *Sphagnum compactum*, N – *Sphagnum cuspidatum*, O – *Sphagnum magellanicum*, P – *Sphagnum papillosum*, Q – *Sphagnum subnitens*, R – *Trichophorum germanicum*, S – *Ulex europaeus*.


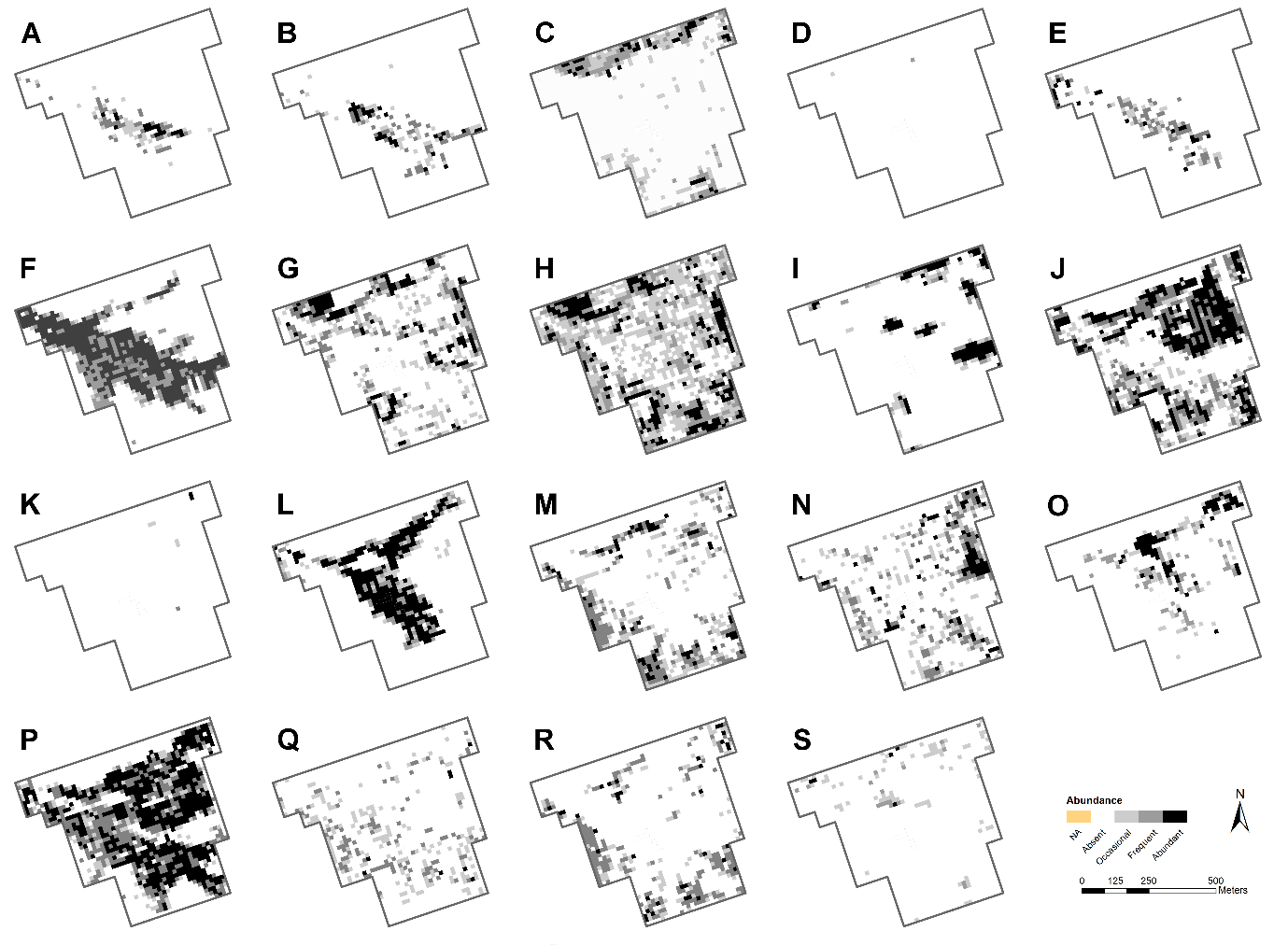


**Figure S4**. Map showing the distribution of species mapped in 2016. A – *Carex rostrata*, B – *Cirsium dissectum*, C – *Erica cinerea,* D – *Lycopodiella inundata,* E – *Menyanthes trifoliata*, F – *Myrica gale*, G – *Pinus sylvestris* (sapling), H – *Pinus sylvestris* (seedling), I – *Pteridium aquilinum*, J – *Rhynchospora alba*, K – *Rhynchospora fusca*, L – *Schoenus nigricans*, M – *Sphagnum compactum*, N – *Sphagnum cuspidatum*, O – *Sphagnum magellanicum*, P – *Sphagnum papillosum*, Q – *Sphagnum subnitens*, R – *Trichophorum germanicum*, S – *Ulex europaeus*.


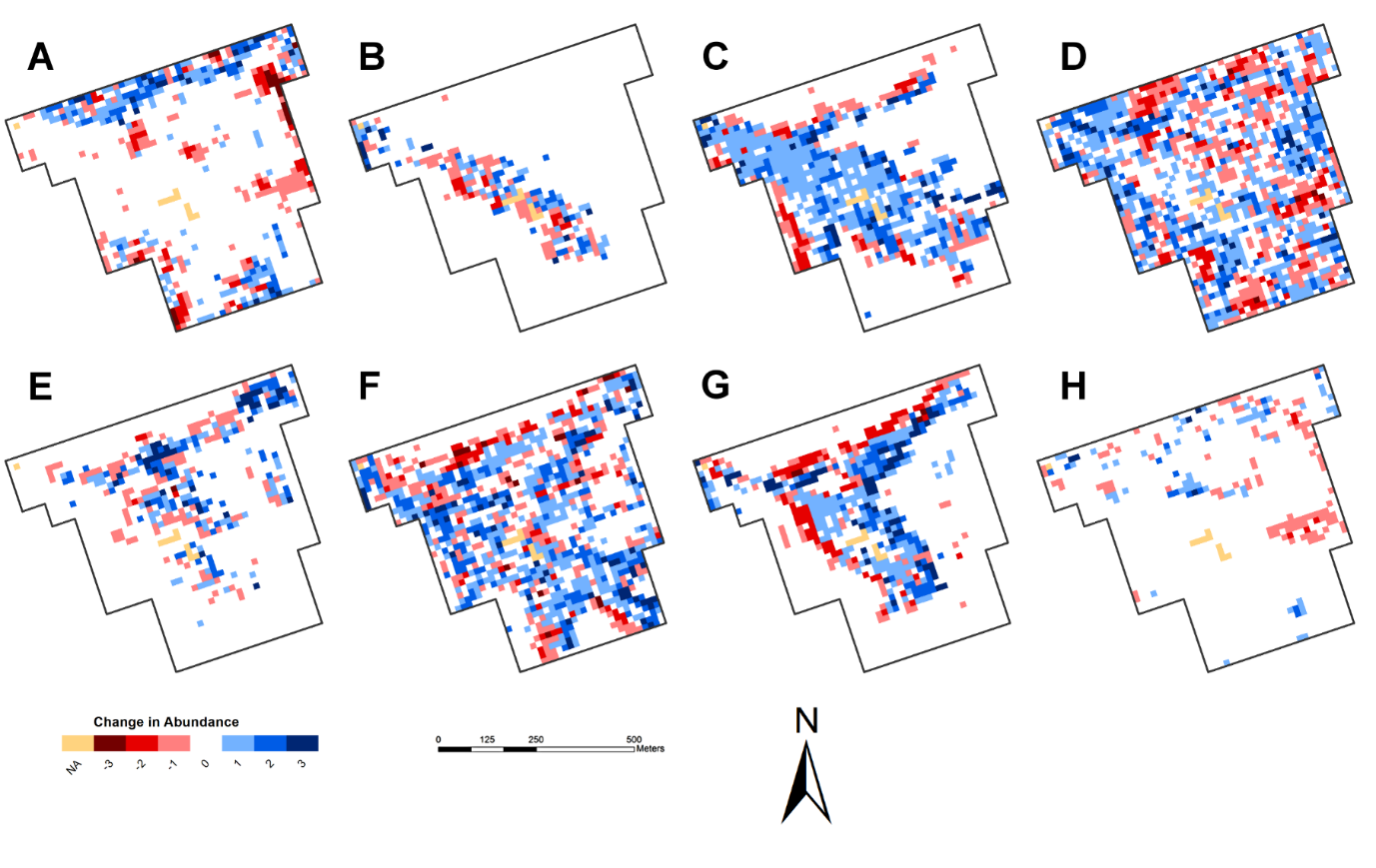


**Figure S5**. Maps showing distribution changes in species that did not differ significantly in area of occupancy between 1951 and 2016. A – *Erica cinerea*, B – *Menyanthes trifoliata*, C – *Myrica gale*, D – *Pinus sylvestris* (seedling), E – *Sphagnum magellanicum*, F – *Sphagnum papillosum*, G – *Schoenus nigricans*, H – *Ulex europaeus*. The pixels labelled NA were those that could not be visited during the 2016 resurvey.


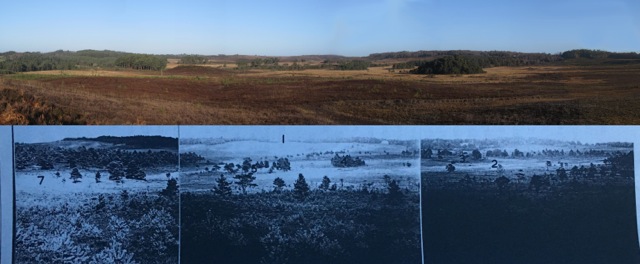


**Figure S6.** Photographs of Cranes Moor, taken from the southern edge, looking towards the north. The upper image was taken in 2016, and the lower images were taken by Palmer Newbould in 1951 (see Newbould 1953). Note the copious pine regeneration in images from both dates; pine is routinely removed from the area through management interventions such as burning and cutting. However some locations (such as the hillock near the label ‘3’ in the lower image) have become increasingly covered by mature trees over the survey interval. The site is heavily browsed by livestock and deer, and the intensity of herbivory has likely increased significantly since 1951; however animals appear to avoid the wetter parts of the site, which is located towards the centre of the image.

**References**

Newbould, P. J. (1953). *The ecology of Cranesmoor*. PhD thesis, University College, London.
